# What is the uptake rate of breast self-examination in Iranian women? Estimation based on systematic review and meta-analysis

**DOI:** 10.1186/s12905-023-02688-3

**Published:** 2023-10-17

**Authors:** Bahman Ahadinezhad, Omid Khosravizadeh, Sima Rafiei, Nastaran Habibi, Zahra Karimkhani, Aisa Maleki

**Affiliations:** 1 Department of Health Services Management, Social Determinants of Health Research Center, Research Institute for Prevention of Non-Communicable Diseases, Qazvin University of Medical Sciences, Qazvin, Iran; 2 Qazvin University of Medical Sciences, Qazvin, Iran; 3 Department of Health Services Management, Student Research Committee, Qazvin University of Medical Sciences, Qazvin, Iran

**Keywords:** Breast self-examination (BSE), Meta-analysis, Iran

## Abstract

**Background:**

Breast self-examination is a simple, painless, confidential and inexpensive screening method for early diagnosis that does not require specialized tools and equipment. In this study, we have estimated the pooled percentage of breast self-examination (BSE) in Iranian women.

**Methods:**

All the published literatures between 2012 and 2022 have been reviewed. Searches were performed in PubMed, Web of Science, ProQuest, Science Direct, Google Scholar, Scientific Information and Magiran databases. The effect size was the pooled percentage of breast self-examination (BSE). In order to check the heterogeneity, the estimation of the I^2^ index and extraction of the Galbraith plot were used, and the drivers of heterogeneity have been identified through meta-regression and estimates were made based on subgroups. All the analysis was done in STATA 15.

**Results:**

From the initial 294 records, 38 were included in the final analysis in which 9960 women have been studied. The heterogeneity of the studies was high based on the variation in OR (I^2^ = 98.4%, heterogeneity X^2^ = 2278.21 (d.f. = 37), *p* < 0.01). The pooled rate of BSE based on fixed and random methods was obtained as 15.46 (95% CI: 14.83 to 16.09) and 24.74 (95% CI: 19.62 to 29.86) percent, respectively. The highest pooled percentage BSE (39.41%, 95% CI: 30.98 to 47.83) was obtained from studies that investigated the action phase in the Trans theoretical model. The pooled percentage obtained from the studies conducted in the central regions of Iran was higher than other cities (27.47%, 95% CI: 17.38 to 37.55).

**Conclusion:**

The result from our analysis determined that performing breast self-examination in Iranian women is low. Health policy makers can increase the rate of breast self-examination in Iran by implementing basic educational programs in schools and encouraging and justifying women in social health centers.

## Introduction

Based on the estimate, 2,261,419 new cases of breast cancer occurred in 2020 [1]. Also, breast cancer is the fifth cause of cancer-related death in women worldwide [1, 2]. Globally, the incidence of breast cancer increased to 2,002,354 in 2019. Also, this year, global mortality and DALYs from breast cancer increased to 700,660 and 20,625,313, respectively [3]. Breast cancer is the leading female cancer in Asia and its incidence has been continuously increasing in the last three decades [4–6]. According to estimates by Sharma [4], the incidence of female breast cancer in Asia has increased from 245,045 in 1990 to 914,878 in 2019. The number of deaths has more than doubled. For the early detection of breast cancer, the World Health Organization [7] recommends mammography, but notes that this method is not affordable in countries with limited medical resources due to its high cost. Mammography and clinical examination are recommended for people who are at higher risk [8]. One of the cost-effective methods of breast cancer screening is self-examination. Breast self-examination is a simple, painless, confidential and inexpensive screening method for early diagnosis that does not require specialized tools and equipment [9]. This procedure is a low-cost, non-invasive and time-saving method and can be performed even by women at home [10]. Also, the Breast Health Global Initiative guidelines recommend BSE as the first breast cancer prevention measure in low- and middle-income countries [11]. There is evidence that regular breast self-examination is positively connected with early detection of breast cancer [12]. Despite the seeming benefits of breast self-examination in countries with a lack of health resources, the uptake rate of it is low. Since Breast self-examination is necessary for the early detection of breast cancer and timely initiation of treatment, its rate should always be monitored. Therefore, this systematic review and meta-analysis have been conducted with the aim of investigating the rate of Breast self-examination uptake in Iranian women.

## Materials and methods

### Search strategy

This research was accomplished based on PRISMA guidelines. To obtain relevant evidence, papers registered from January 1, 2012, to September 11, 2022, have been systematically reviewed. The search strategy was designed as follows and documents were searched using Booleans in the title, abstract and keywords. Search strategies according to databases are presented in Table 1.

1. Breast
2. Self-examin*
3. BSE
4. Iran
  = ((1 & 2) OR 3) & 4

**Table 1:** Search strategies

Data base	Search strategy	#
**PubMed**	((Breast[Title/Abstract] AND self-examin*[Title/Abstract]) OR (BSE[Title/Abstract])) AND (Iran[Title/Abstract] AND (2012/1/1:2022/9/13[pdat])) Filters: English, Persian, from 2012/1/1—2022/9/13	46
**ProQuest**	noft(((Breast AND self-examin*) OR BSE)) AND noft(Iran) Additional limits—Date: From January 01 2012 to September 13 2022 English Articles	23
**WOS**	(TI = (((Breast AND self-examin*) OR BSE) AND Iran)) OR AB = (((Breast AND self-examin*) OR BSE) AND Iran) Refined By:Document Types: Article or Proceeding Paper or Review Article.Languages: English	47
**Science Direct**	Title, abstract, keywords: ((Breast AND self-examination) OR BSE) AND Iran 2012–2023 Research articles Review articles	8
**Google Scholar**	(Breast AND self-examin*) OR BSE) Universities of medical sciences’ names 2012–2022	63
**Sid.ir**		30
**Magiran**		77
**Total**		**294**

**Fig. 1:**
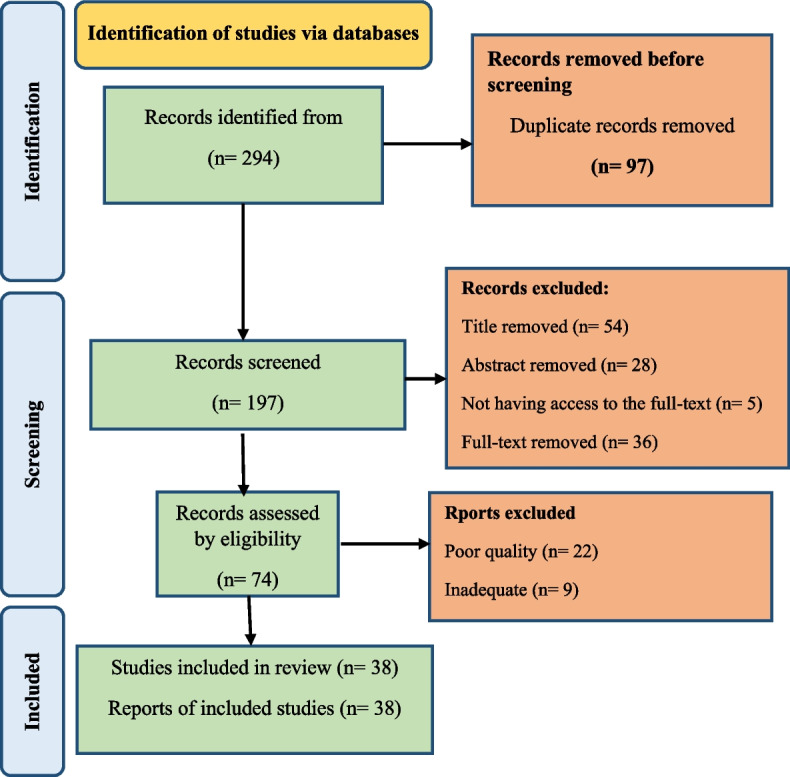
PRISMA flowchart

Figure 1 displays the PRISMA flowchart. In the initial search, 294 articles were found. After the necessary screenings, 38 studies were included in the analysis.

### Eligibility criteria

We included studies that met the following eligibility criteria: 1) published in Persian and English, 2) conducted in Iranian population, 3) having a quantitative design, 4) published during the period from 2012 to 2022, 5) used only human samples and 6) had the data needed to calculate the effect size, and 7) narratives and qualitative studies were excluded.

### Data extraction

Two researchers extracted data using a structured checklist. The following information was extracted from each study: name of the authors, year of publication of the paper, place of the study, self-examination measure, studied population, sample size, proportion of self-examination and standard deviation.

### Literature quality assessment

The quality assessment was done by two people so that each person evaluated the studies separately and independently based on the STROBE Checklist of cross-sectional studies. The tool used was the STROBE checklist used to assess the quality of observational studies. STROBE contains 22 components that cover different parts of the article [13]. Articles that showed more than 50% compliance with the checklist were included in the study.

### Maine outcome

In this study, the pooled percentage of breast self-examination (BSE) is main outcome.

### Data analysis process

In the first step, the data of eligible studies have been entered into an Excel file. Then, pooled percentage of BSE has been estimated using fixed effects model. Next the amount of heterogeneity was evaluated based on I^2^ index. Considering the significance of the heterogeneity, in the final step, the pooled percentage of BSE was estimated using the random effects method. The confidence interval of the estimates was considered to be 95%. All the analysis was completed in STATA 15.

### Assessment of heterogeneity

In order to check the heterogeneity, the I^2^ index has been estimated and the Galbraith plot extracted, and the drivers of heterogeneity have been identified through meta-regression estimation.

### Assessment of publication bias

The presence of publication bias was checked graphically by funnel plot, by nonparametric trim-and-fill analysis and by egger test.

### Sensitivity analysis

The sensitivity of the pooled effect size to the results of each study was assessed by using leave-one-out meta-analysis.

## Results

Out of the initial 294 records, 38 studies were included in the meta-analysis. The total number of women who were examined in the meta-analyzed studies was 9960. The Table 2 shows the characteristics of the studies included in the analysis.

**Table 2:** Characteristics of studies included in the final analysis

Ref	Study	Uptake measure	Year	City	Studied population	n
[14]	Absavaran et al	Performing regularly BSE	2015	Zabol	Nurses	35
[15]	Akhtarizavare et al	Performing BSE	2014	Hamedan	Women visiting the healthcare center	384
[16]	Amiri et al	Performing regularly BSE	2021	Sari	Women visiting the healthcare center	279
[17]	Askarimajdabadi et al	Performing regularly BSE	2020	Aq Qala	Healthcare employees	261
[18]	Bashirian et al	Performing regularly BSE	2021	Hamadan	University employees	44
[19]	Bashirian et al	Performing regularly BSE	2019	Hamadan	University employees	501
[20]	Didarloo et al	Performing BSE	2017	Urmia	Medical Sciences Students	334
[21]	Farshbafkhalili et al	Performing BSE	2014	Tabriz	Women visiting the healthcare center	400
[22]	Fayazi et al	Performing BSE	2013	Ahvaz	Students	237
[23]	Ghasemi et al	Performing BSE	2014	Shahrekord	University employees	50
[24]	Haghighi et al	Performing BSE	2015	Birjand	University employees	89
[25]	Hajiantilaki & Auladi	Performing regularly BSE	2012	Babol	Women visiting the healthcare center	500
[26]	Hajmahmoodi et al	Performing regularly BSE	2002	Tehran	Healthcare employees	410
[27]	Hasani et al	Performing regularly BSE	2011	Bandarabbas	Women visiting the healthcare center	240
[28]	Irandoost et al	Performing BSE	2020	Tehran	House wives	859
[29]	Mahmoodi & Ramazani	Performing regularly BSE	2011	Zabol	Women visiting the healthcare center	246
[30]	Mashhodkermanchi et al	Performing BSE	2018	Tehran	Women visiting the healthcare center	47
[31]	Matlabi et al	Performing regularly BSE	2021	Gonabad	Women visiting the healthcare center	70
[32]	Matlabi et al	Performing BSE	2018	Gonabad	Women visiting the healthcare center	70
[33]	Matlabi et al	Action in Transtheoretical Model	2018	Gonabad	Women visiting the healthcare center	70
[34]	Miri et al	Action in Transtheoretical Model	2020	Birjand	House wifes	450
[35]	Mirsafi et al	Performing BSE	2021	Shazand	Women visiting the healthcare center	16
[36]	Momenyan et al	Performing BSE	2014	Qom	Midwifery and nursing students	113
[37]	Moodi et al	Action in Transtheoretical Model	2019	Birjand	Women visiting the healthcare center	450
[38]	Morowatisharifabad et al	Performing regularly BSE	2019	Yazd	Patients with Breast Cancer	159
[39]	Movahed et al	Performing regularly BSE	2011	Shiraz	Students	305
[40]	Neinavaie et al	Performing BSE	2017	Karaj	Women visiting the healthcare center	200
[41]	Paknejad & saeedi	Performing regularly BSE	2019	Tehran	House wifes	220
[42]	Parsa et al	Performing BSE	2016	Hamadan	Women visiting the healthcare center	75
[43]	Pilehvarzadeh et al	Performing regularly BSE	2014	Jiroft	Women visiting the healthcare center	200
[44]	Pirasteh et al	Action in Transtheoretical Model	2012	Tehran	Women visiting the healthcare center	302
[45]	Pirzadeh	Action in Transtheoretical Model	2018	Isfahan	Medical Sciences Students	384
[46]	Reisi et al	Performing regularly BSE	2013	Isfahan	Healthcare employees	119
[47]	Rezabeigidavarani et al	Performing regularly BSE	2016	Kerman	Women visiting the healthcare center	300
[48]	Rokhforouz et al	Action in Transtheoretical Model	2019	Rafsanjan	Health volunteers	46
[49]	Sahraee et al	Performing regularly BSE	2013	Bushehr	Women visiting the healthcare center	400
[50]	Shakery et al	Performing regularly BSE	2021	Jahrom	Women visiting the healthcare center	75
[51]	Zaremarzouni et al	Performing BSE	2015	Dezful	Women visiting the healthcare center	1020

**Fig. 2:**
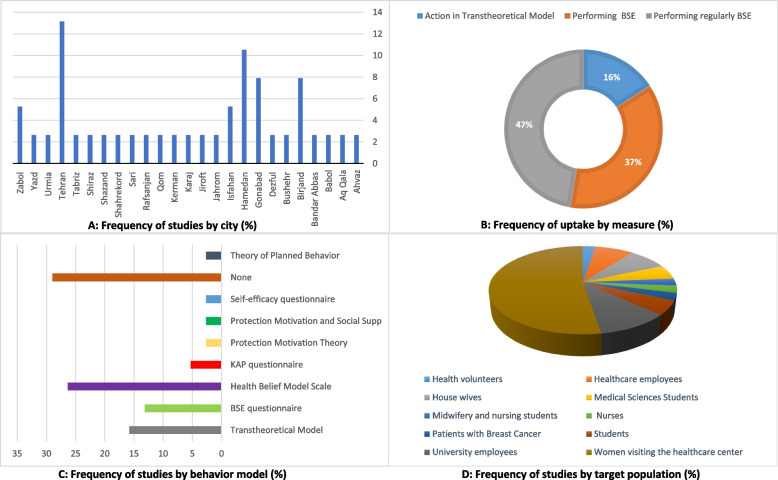
Frequency description of the reviewed studies by heterogeneity factors

Figure 2-a, displays the frequency distribution of reviewed studies in the Iran. Most of the studies (About 13%) have been done in Tehran. Also, Hamadan (About 10%), Birjand (About 8%) and Gonabad (About 8%) are in the next ranks. Based on Fig. 2-b, about 50 percent of the studies have questioned regular breast self-examination, 37 percent investigated performing self-examination, and 16 percent questioned the action stage in the Transtheoretical Model. Figure 2-c demonstrates that about 29 percentage of the studies did not use a specific model. While the health belief model has been used in 26 percent of studies. Also, the Transtheoretical Model (15.8%) has been ranked next. Based on Fig. 2-d, about 53 percent of the articles have been studied by women referring to the health center. Employees (18.5%) and students (10.5%) are next.

### Assessment of heterogeneity size

**Fig. 3:**
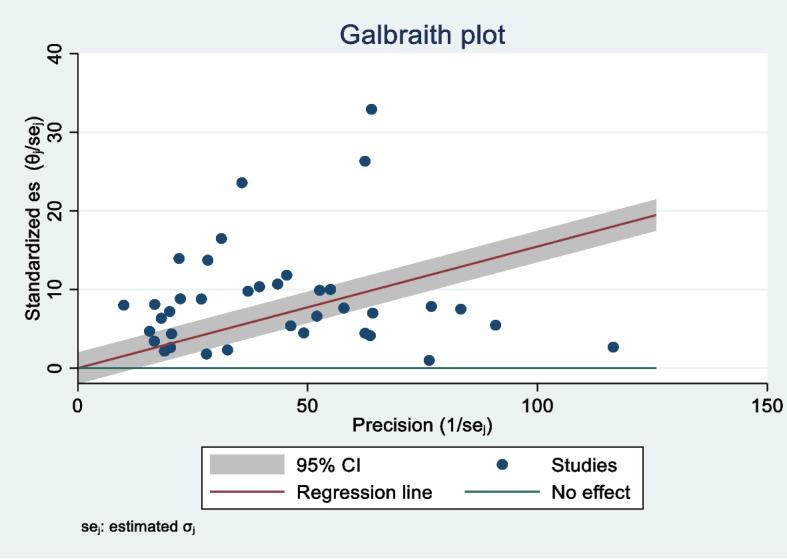
Galbraith plot based on fixed and random effect models

The information obtained from the output of the software revealed that the analyzed studies have considerable heterogeneity. So that value of I^2^ index 98.4% was found (heterogeneity X^2^ = 2278.21, d.f. = 37, *p* < 0.01). Meta regression results determined heterogeneity factors. Differences in data gathering tool, measure of breast self-examination uptake, studied population and place of study (city) have been the main sources of heterogeneity between studies (*p* < 0.01). Figure 3 illustrates the heterogeneity checking through the Galbraith plot. Since there is no dot on the green line, the percentage of breast self-examination was not zero in any of the studies. The slope of red line equals the estimate of the pooled BSE uptake percent, which is equal to 24.74 (95% CI: 19.62 to 29.60). Given that 27 out of the 38 studies were outside the 95% CI region, thus there is considerable heterogeneity among the studies.

### Pooled percentage of breast self-examination (BSE) uptake

In Table 3, the pooled percentage of breast self-examination (BSE) uptake according to fixed and random effects models is presented. Based on the fixed effects method, the pooled percentage of BSE uptake. The pooled rate of BSE based on fixed and random methods was obtained as 15.46 (95% CI: 14.83 to 16.09) and 24.74 (95% CI: 19.62 to 29.86) percent, respectively.

**Table 3:** Effect size (Pooled odds ratio) based on fixed and random effect models

Test	BSE %	95% CI
Fixed Effect	15.46	14.83 to 16.09
Random Effect	24.74	19.62 to 29.86
*BSE *Breast self- examination		

**Fig. 4:**
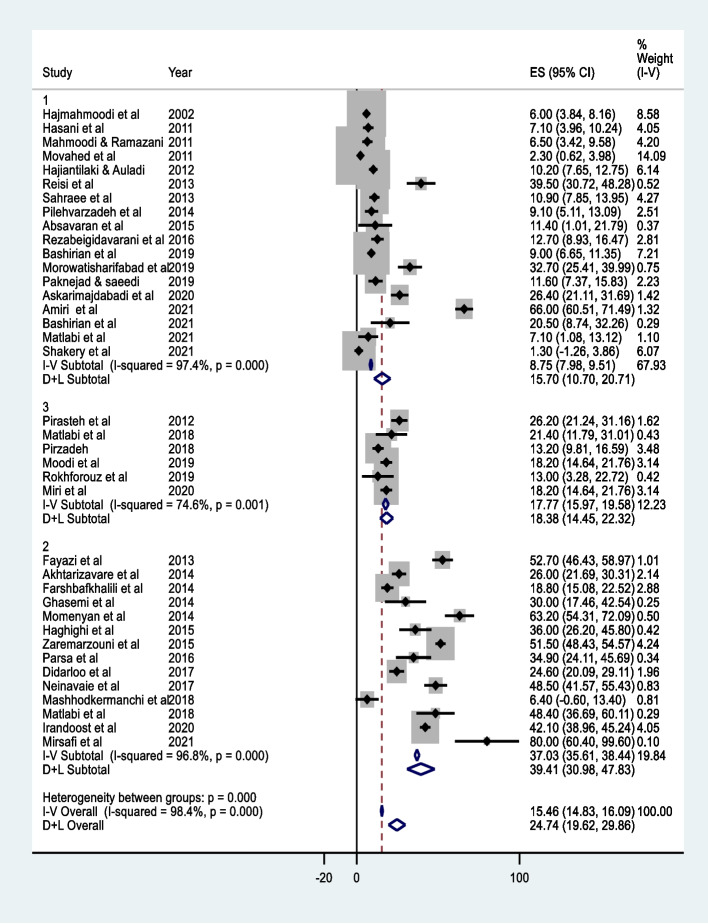
Forest plot based on fixed and random effects models (by BSE measure), 1) Performing regularly BSE, 2) Performing BSE, 3) Action in Trans theoretical Model

Because of the high heterogeneity among studies, the pooled percentage of breast self-examination was estimated by factors such as breast self-examination measure, population and location. Figure 4 displays the forest plot by the breast self-examination measure. The highest pooled percentage of breast self-examination (39.41%, 95% CI: 30.98 to 47.83) was obtained from studies that investigated the action phase in the Trans theoretical model. On the other hand, the pooled percentage of BSE in studies investigating regular breast self-examination is the lowest value (15.70%, 95% CI: 10.70 to 20.71).

**Fig. 5:**
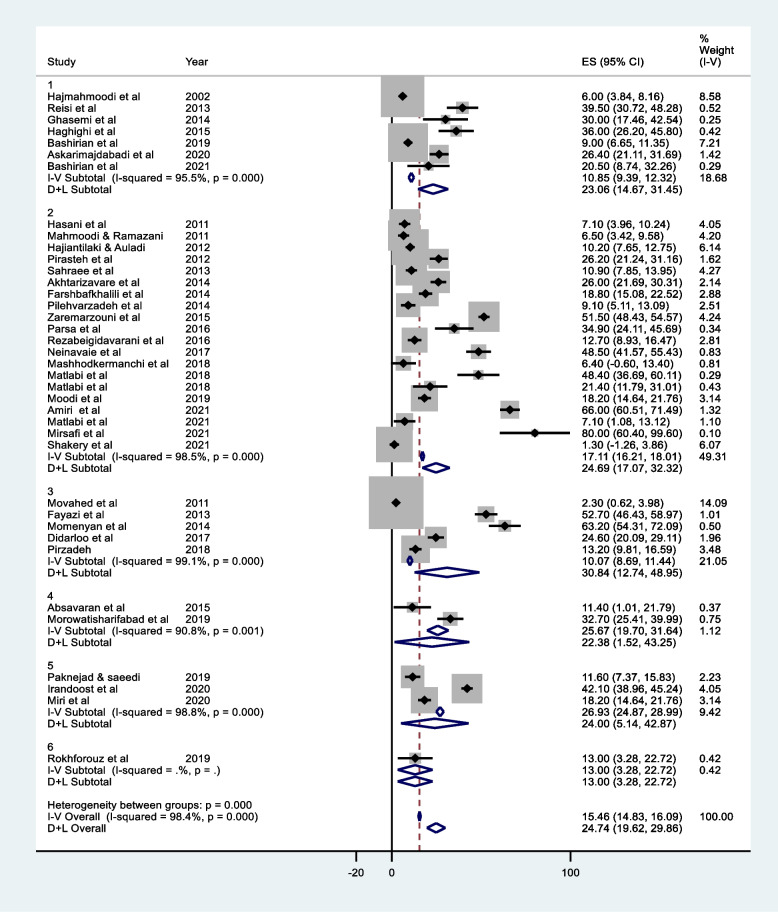
Forest plot based on fixed and random effects models (by study population), 1) Healthcare employees, 2) Women visiting the healthcare center, 3) Medical Sciences Students, 4) Nurses, 5) House wives, 6) Health volunteers

Figure 5 shows the Forest plot by the studied groups. The highest percentage of breast self-examination (30.84%, 95% CI: 12.74 to 48.95) has been gained for medical students. However, the percentage of breast self-examination among health volunteers was the lowest (13.00%, 95% CI: 3.28 to 22.72). Also, the pooled percentage of BSA in medical students was higher than other groups (30.84%, 95% CI: 12.74 to 48.95).

**Fig. 6:**
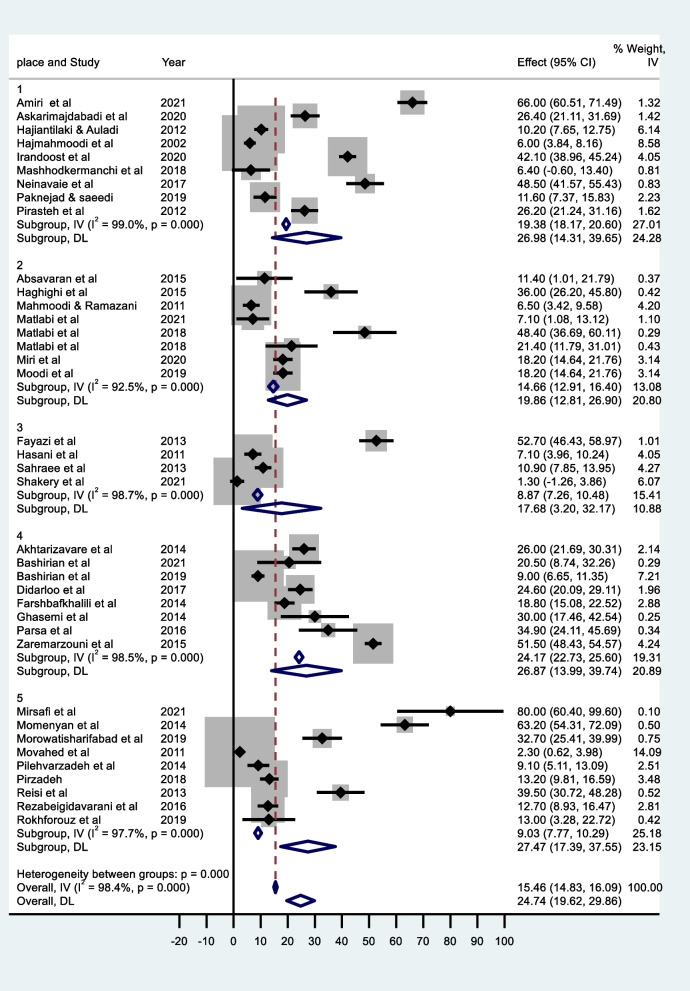
Forest plot based on fixed and random effects models (by study population), 1) Northern cities, 2) Eastern cities, 3) Southern cities, 4) Western cities, 5) Central cities

In Fig. 6, the Forest diagram of the pooled percentage of breast self-examination based on the geographical regions of Iran displayed. The highest percentage of self- examination (27.47%, 95% CI: 17.38 to 37.55) is reported for central cities. While the rate of this screening action was the lowest (17.68%, 95% CI: 3.19 to 32.17) in southern cities.

### Assessment of publication bias

**Fig. 7:**
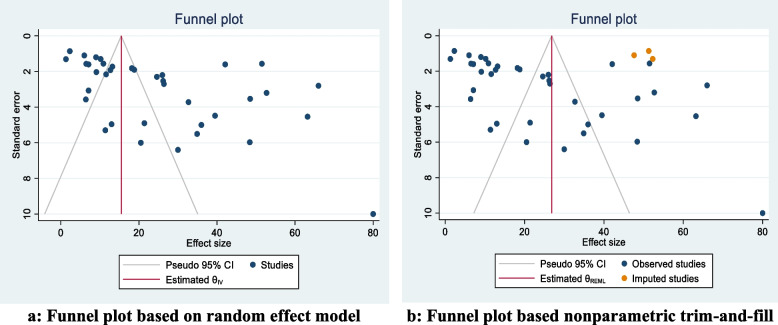
Checking the existence of publication bias based on funnel plot. **a** Funnel plot based on random effect model. **b** Funnel plot based nonparametric trim-and-fill

The results of the Egger test confirm the small study effects (*p* < 0.01). As well as the publication bias assessment are presented graphically by funnel plot in Fig. 7 by random effect-based funnel plot (Fig. 7-a) and nonparametric trim-and-fill analysis in Fig. 7-b. In the funnel plot, the studies are almost asymmetrically distributed, and most of the studies are located at the top of the funnel (that is, studies with high precision) but out of the 95% confidence interval. The plot obtained reveals the existence of the publication bias. Based on plot b in Fig. 7 the three studies (orange dots) trimmed and filled on the right side of the funnel plot can be attributed to the possible presence of publication bias. Also, based on the information in Table 4, it is clear that imputing 3 studies (orange dots) on the right side of the funnel plot could lead to an increase in the pooled percentage of BSE from 24.89 (95% CI: 18.86 to 30.92) to 26.84 (95% CI: 20.86 to 32.81).

**Table 4:** Nonparametric trim-and-fill analysis of publication bias, imputing on the right and left

Imputing Side	Studies	Pooled rate	[95% conf. interval]
Imputing on the left	Observed	24.89	18.86 to 30.92
	Observed + Imputed	24.89	18.86 to 30.92
Imputing on the right	Observed	24.89	18.86 to 30.92
	Observed + Imputed	26.84	20.86 to 32.81

### Sensitivity analysis

By performing the Leave-one-out meta-analysis, we evaluated the sensitivity of the pooled percentage of BSE uptake to the role of individual studies. Leave-one-out analysis shows that excluding individual studies causes to vary the pooled BSE percent. So that the elimination of Shakery et al. (2021) study reduces the pooled BSE to 23.67 (95% CI: 18.00 to 29.34) percentage. While excluding the study of Zaremarzouni et al. (2015), the percentage of breast self-examination increases to 25.54% (95% CI: 19.48 to 31.60).

## Discussion

Studies conducted in Iran have reported different levels of breast self-examination. For this reason, the present study was done with the aim of estimating the pooled percentage of this cost-effective preventive action. In order to reach the final rate of breast self-examination, the researchers systematically reviewed the evidence published during the period 2012 to 2022 and meta-analyzed the percentage of up taking this preventive behavior. The investigation carried out in this research determined that all studies have measured the rate of breast self-examination by asking one question (in the form of self-report by women).

Our estimation disclosed that the pooled rate of breast self-examination in Iranian women is about 24.74 percent (95% CI: 19.62 to 29.86). The rate of performing this screening varied from 1.3 percentage in Shakery et al. [50] study to 80 percent in Mirsafi et al. [35] work. The first study was conducted in Jahrom and among women visiting the health center. Researchers have attempted to find the percentage of regular breast self-examination by using the health belief model. In the second study, which was conducted on women visiting the health center in Shazand, the investigators have calculated the percentage of breast self-examination using the self-efficacy model.

Since our study is the first meta-analysis of breast self-examination percentage in Iran, for this reason we are not able to compare our estimate with other studies. However compared to the findings of the study conducted in Vietnam (15.2%), the pooled rate of breast self-examination was gained higher in our study [52].

Almost close to our result, in another meta-analysis, the authors estimated the pooled breast self-examination rate among Ethiopian women to be 36.72 percent (95% CI: 29.90 to 43.53) [53]. But in meta-analysis of Mekonnen [54], the percentage of breast self-examination in Ethiopia was obtained almost double our estimate. In the mentioned study, after reviewing 9605 studies, the authors included 12 studies including 4129 health workers in the meta-analysis. Eventually the pooled prevalence of breast self-examination practice among health care workers was estimated to be 56.31 percent (95% CI: 44.37 to 68.25). While the analysis of our subgroup showed that the pooled rate of breast self-examination among health workers in the studies conducted in Iran is about 23 percent (95% CI: 14.67 to 31.45).

In another meta-analysis in Africa, 56 studies with a total of 19,228 participants were included in the final analysis. The pooled prevalence of breast self-examination in Africa was 44.0% (95% CI: 36.63 to 51.50) and 17.9% (95% CI: 13.36 to 22.94), respectively [55]. Differences in estimates could be due to differences in health programs among countries and methodological factors among studies. For example, some countries may start education and promoting breast self-examination from high school in the form of a basic health program**.** On the other hand, the studies included in other meta-analyses may be different from our analysis in terms of data collection tools, target group, and study design. Due to the diversity of target groups, data collection tools and locations in the studies included in the analysis, we estimated the pooled percentage of breast self-examination based on these differences.

Based on our findings, the highest percentage of self- examination (27.47%, 95% CI: 17.38 to 37.55) is reported for central cities. While the rate of this screening action was the lowest (17.68%, 95% CI: 3.19 to 32.17) in southern cities. Various socio-economic, psychological and contextual factors can explain this difference [56]. Regional differences in the pooled percentage of breast self-examination have also been shown in other studies. For example, Seifu & Mekonen, [55] indicated that the percentage of performing this action has a statistically significant difference between African regions. In the subgroup analysis, there was a significant difference between the highest performing sub regions in West Africa, 58.87% (95% CI: 48.06 to 69.27) and the lowest in South Africa, 5.33% (95% CI: 2.73 to 10.17). Another part of our findings exhibited that the percentage of breast self-examination is dissimilar among different population groups. So that the highest percentage of breast self-examination (30.84%, 95% CI: 12.74 to 48.95) has been gained for medical students. However, the percentage of breast self-examination among health volunteers was the lowest (13.00%, 95% CI: 3.28 to 22.72). The observed difference has also been reported by other studies [54, 57]. The high percentage of breast self-examination in medical science students compared to other groups can be explained by the fact that having knowledge can increase people's risk perception, and this makes health science students perform breast self-examination.

Our analysis revealed that the percentage of breast self-examination in Iranian women is low. Various determinants can be the reason for the low rate of breast self-examination among Iranian women. Women's breast health behavior can depend on factors such as: health policy context, socio-economic status, cultural, psychological and behavioral factors [58]. For example, the findings of a study in Iran showed that perceived barriers, knowledge and level of education were related to BSE. The results of Dewi et al. [59] study in Surabaya indicated that breast cancer knowledge and attitudes toward BSE were associated with performing BSE. Also, perceived benefits and barriers and subjective norms were significantly related to the intention and doing of BSE.

On the other hand, previous studies [60, 61] support the hypothesis that Reasoned Action Approach and Health Belief Model components are important in predicting the up taking of BSE. In another study, researcher found that women who were younger, with a higher level of education, had fewer children and were employed, were more aware of breast self-examination and requested it [62]. Although BSE is not recommended by WHO as a screening method, it can be used as a measure to raise awareness of women at risk [63]. This examination method can be useful in settings where the economic power of women is low and there is no effective access to more advanced diagnostic procedures. As expected, the pooled percentage of breast self-examination was higher than performing regularly it. This difference can be due to self-control problems, procrastination and other factors affecting behavioral compliance.

The studies that we included in the meta-analysis mainly measured breast self-examination by asking people and their self-reports. The self-report method suffers from certain disadvantages due to the behavior of the general public. Self-reported responses may be exaggerated. Various biases such as social desirability bias may affect the results. Also, people may forget relevant details. Self-report instruments can be influenced by the person's emotions at the time of filling out the questionnaire.

The results of this study should be generalized with caution. Because the studies included in the analysis had limitations such as the non-optimal volume and sampling method, the use of self-expression of people. It is better to conduct future survey studies in the field of breast self-examination by removing this limitation.

## Conclusion

The result obtained from our analysis determined that performing breast self-examination in Iranian women is low. Compared to other developed and developing countries, this rate was less. As discussed, various socio-economic, psychological and contextual factors can explain this difference. Health policy makers can increase the rate of breast self-examination in Iran by implementing basic educational programs in schools and training sessions for women in health care centers.

## Data Availability

All data generated or analyzed during this study are included in this article.
